# The effect of motor resource suppression on speech perception in noise in younger and older listeners: An online study

**DOI:** 10.3758/s13423-023-02361-8

**Published:** 2023-08-31

**Authors:** Kate Slade, Alanna Beat, Jennifer Taylor, Christopher J. Plack, Helen E. Nuttall

**Affiliations:** 1 Neuroscience of Speech and Action Laboratory, Department of Psychology, Lancaster University, Lancaster, UK; 2 Lancaster Medical School, Lancaster University, Lancaster, UK; 3 Manchester Centre for Audiology and Deafness, School of Health Sciences, University of Manchester, Manchester, UK

**Keywords:** Ageing, Speech perception, Speech motor, Hearing loss, Auditory-motor

## Abstract

Speech motor resources may be recruited to assist challenging speech perception in younger normally hearing listeners, but the extent to which this occurs for older adult listeners is unclear. We investigated if speech motor resources are also recruited in older adults during speech perception. Specifically, we investigated if suppression of speech motor resources via sub-vocal rehearsal affects speech perception compared to non-speech motor suppression (jaw movement) and passive listening. Participants identified words in speech-shaped noise at signal-to-noise ratios (SNRs) from -16 to +16 dB in three listening conditions during which participants: (1) opened and closed their jaw (non-speech movement); (2) sub-vocally mimed ‘the’ (articulatory suppression); (3) produced no concurrent movement (passive listening). Data from 46 younger adults (M age = 20.17 years, SD = 1.61, 36 female) and 41 older adults (M age = 69 years, SD = 5.82, 21 female) were analysed. Linear mixed effects modelling investigated the impact of age, listening condition, and self-reported hearing ability on speech perception (d’ prime). Results indicated that speech perception ability was significantly worse in older adults relative to younger adults across all listening conditions. A significant interaction between age group and listening condition indicated that younger adults showed poorer performance during articulatory suppression compared to passive listening, but older adults performed equivalently across conditions. This finding suggests that speech motor resources are less available to support speech perception in older adults, providing important insights for auditory-motor integration for speech understanding and communication in ageing.

## Introduction

Across the UK, hearing loss affects approximately 70% of people aged 70+ years (Royal National Institute for Deaf People (RNID), 2020). A major complaint of people with age-related hearing loss (ARHL) is that they struggle to understand speech in noisy environments (Ward et al., 2017). Perceiving speech in noise may require increased ‘listening effort’, meaning that increased neural or cognitive resources are needed to hear successfully (Pichora-Fuller et al., 2016). If communication is too effortful, adults with ARHL may withdraw from social situations, leading to loneliness (Shukla et al., 2020). Importantly, both hearing loss and social isolation are associated with increased risk for dementia (Brewster et al., 2020; Dawes et al., 2015; Livingston et al., 2020). Investigating the use of neural resources during speech perception is essential for understanding how ageing and associated hearing difficulties affect neural functioning and brain health.

Older adults with hearing loss show differences in functional brain activity across auditory, sensory and motor domains compared to those without hearing loss (for reviews, see Griffiths et al., 2020; Slade et al., 2020). However, in this paper, we focus on age- and hearing-related differences in the use of motor resources during speech perception. Neuroscientific studies first established a potential role for motor activity during low difficulty speech perception. For example, studies employing transcranial magnetic stimulation (TMS) and associated motor evoked potentials (Fadiga et al., 2002; Watkins et al.,^ 2003^) and functional magnetic resonance imaging (fMRI; Wilson et al., 2004) showed that neural excitability increases in the articulatory motor cortex during speech perception in quiet. Evidence then emerged to suggest that motor activity is increased during difficult speech perception in younger adults in studies employing TMS (Adank, 2012; D’Ausilio et al., 2012; Nuttall et al., 2016, 2017) and fMRI (Du et al., 2014).

The role of the articulatory motor activity in speech perception is debated (Hickok et al., 2011; Stokes et al., 2019). Nuttall et al. (2016) found that increases in motor activity were associated with better perception of distorted speech in younger adults. Further, supressing neural activity in the motor cortex, using disruptive, repetitive TMS, negatively affects the ability to perceive ambiguous speech in younger listeners (Rogers et al., 2014; Smalle et al., 2015) and during phoneme discrimination (Meister et al., 2007). Stokes et al. (2019) employed a dual task wherein younger adults identified phonemes whilst performing an articulation task to suppress speech motor resources. Phoneme identification was measured during four conditions: (1) articulatory suppression, in which participants sub-vocally repeated the word “*the*” every second; (2) mandible movement, in which participants opened and closed their jaw every second; (3) foot tapping, in which participants tapped their foot every second; and (4) passive listening, in which participants did no additional movement task. Phoneme identification was poorest during articulatory suppression, compared to the movement control tasks and passive listening, in younger listeners. The authors concluded that the speech motor system may play a minor role in speech perception.

However, it is less clear how articulatory motor resources contribute to speech perception in older listeners. If increased activation of articulatory motor resources is functional for challenging listening, then older adults may also use these resources to compensate for age-related auditory impairments. This is known as the ‘Motor Compensation Hypothesis’: Speech motor resources are up-regulated to assist with speech perception after age-related damage to peripheral auditory processing. Supporting fMRI evidence finds that older adults show increased activity in speech motor areas during listening, which correlated with better speech perception (Du et al., 2016). Alternatively, evidence from TMS studies finds that older adults with hearing loss show reduced speech motor activity during listening compared to older and younger adults without hearing loss (Panouillères & Möttönen, 2018). Further, MRI research indicates that age-related declines in speech perception may be related to reduced volume in premotor cortex, suggesting that auditory-motor interactions may be negatively impacted by ageing (Tremblay et al., 2021). This is known as the ‘Auditory-Motor Decline Hypothesis’: Reduced input to the central auditory system due to hearing loss leads to reduced input to the articulatory motor cortex. This deprivation of input may affect how these resources can be used during speech perception.

## Methods

### Aims and hypotheses

The aim of the current study was to determine the role of articulatory motor resources in speech perception in older adults, who may have poorer auditory function. We conducted a partial replication and extension of a previous study, which employed a dual-task behavioural manipulation to test whether the articulatory motor system provides a compensatory role in speech perception during difficult listening (Stokes et al., 2019). We utilised an adaptation of this experimental paradigm in order to conduct the research online, extending the sample from only younger adults without hearing loss (aged 18–30 years) to older adults (aged 60–85 years) both with and without hearing loss. Our hypotheses were:

- H1. There would be a significant effect of articulatory suppression on speech perception, where speech perception would be poorest during concurrent articulatory motor suppression compared to a movement control condition and passive listening.
- H2. The hypothesised decrease in speech perception performance with greater articulatory suppression will be different for older adults compared to younger adults.
- H3. The change in speech perception performance with greater articulatory suppression for older adults will be predicted by self-reported hearing ability.

This research was pre-registered on the Open Science Framework (OSF) prior to data collection. The pre-registration, experimental code, research data, and analysis scripts can be found online at: https://osf.io/y79n6/.

### Participants

Ninety-two participants were recruited from Lancaster University and the local community. The sample consisted of 46 younger adults (M age = 20.17 years, SD = 1.61, 36 female) and 46 older adults (M age = 69.70 years, SD = 5.94, 21 female). Participants were all self-reported right-handed, monolingual speakers of British English, with normal or corrected-to-normal vision, and without any clinical diagnosis of speech, language, neurological or psychiatric conditions. Nineteen older adults self-reported that they had ARHL; 11 of whom reported wearing hearing aids. Hearing aid wearers removed these for the experiment, and verbally confirmed that they were able to hear well enough without them. Participants also completed the self-report version of the Informant Questionnaire on Cognitive Decline (IQCODE-SR) to pre-screen for cognitive impairment (Jansen et al., 2008). All participants had an IQCODE-SR mean score below 3.65, which is suggested to be an acceptable cut-off for cognitive screening (Jansen et al., 2008). The mean IQCODE-SR score was 2.08 (SD = 0.57) for younger participants and 3.13 (SD = 0.16) for older participants.

Our target sample size was 92 participants, based on an a priori power calculation using the GLIMMPSE package (Kreidler et al., 2013) for linear mixed effects models, in combination with the Superpower application for power simulations (Lakens & Caldwell, 2021); full details of this protocol and related materials can be found in the associated pre-registration https://osf.io/5zyfk. The power calculation assumed .80 power with an alpha of .01, and an effect size of Cohen’s d = .3, which is in-between small and moderate effects (Cohen, 2013). The calculation was based on the power necessary to detect interaction effects between age, task condition and hearing ability, on speech perception. The selected effect size was based on predicted group differences and findings in similar literature (Stokes et al., 2019; Woods et al., 2012, 2015).

### Materials

Participants took part online during a remote Microsoft Teams video call with a member of the research team. The experimental materials were presented to participants using online platforms that controlled the presentation of experimental stimuli and collected participants’ responses: Qualtrics (Qualtrics, Provo, UT) was used to collect self-report responses, and PsychoPy3 (Peirce et al., 2019) hosted via Pavlovia (Bridges et al., 2020) was used to collect behavioural responses from the online speech-perception task. All experimental materials, including stimuli, and experimental scripts can be found online in the OSF repository associated with this research (https://osf.io/y79n6/files/osfstorage).

#### Self-reported hearing ability

Subjective hearing ability was measured using the short version of the Speech and Spatial Qualities of Hearing Scale (SSQ-12) (Noble et al., 2013). The questionnaire consists of 12 items concerning hearing ability in various environments and situations, for example, “*Can you tell how far away a bus or a truck is, from the sound?*” Participants respond on a 10-point Likert scale, where 10 indicates perfect hearing ability, and 0 indicates very poor hearing ability. The final score is the average across all 12 items. The 12-item scale was found to be highly reliable (Cronbach’s alpha = .86 (CI = .81, .90)).

#### Speech perception in noise dual-task

In the speech perception in noise (SPiN) task, participants completed trials in which they identified words presented in background noise. The speech and noise stimuli employed were obtained from the open-source repository (https://osf.io/tqhr5/) associated with a previous study on the role of motor resources during speech perception on which this research was based (Stokes et al., 2019). The speech stimuli consisted of four minimal pairs of synthetic monosyllabic, consonant-vowel-consonant (CVC) English words (“Buy/Pie”, “Die/Tie”, “Buy/Die”, and “Pie/Tie”) originally recorded by a female speaker and subsequently edited to create synthesized versions of each word. The words were embedded in background noise, specifically Gaussian noise filtered to match the long-term average spectrum of the female speaker. For comprehensive details of the speech stimuli and noise characteristics, please refer to the original paper by Stokes et al. (2019).

The speech and noise stimuli obtained from the open-source repository were further edited for use in the online SPiN task. Praat software (Boersma, 2022) was used to embed the speech (429 ms) in the centre of a 1-s segment of the speech-shaped noise. The level of the noise was set at a constant 60 dB SPL. The level of the speech was adjusted to create nine fixed signal-to-noise ratios (SNRs) ranging from -16 dB (difficult) to +16 dB (easier) in 4-dB steps. Each SNR was tested across 24 trials, comprising equal combinations of all four minimal pairs with each pair having six trials per SNR. The SNRs were interleaved, wherein the 24 trials at each SNR level were split into four blocks of six trials, creating a total of 36 blocks across all nine SNR levels. The 36 blocks were presented in a random order. A fixation cross preceded each trial by 0.5 ms, and a response screen followed that displayed two images on either side of the screen corresponding to the meaning of the minimal pairs. The screen location, either left or right, of the image consistent with the presented word was randomised. Participants used their mouse or trackpad to click on the image that corresponded to the word they thought they heard in the trial. The images displayed were: a necktie, a shopping trolley, a slice of pie, and hair dye.

Participants completed the SPiN task under three dual-task conditions: (1) passive listening, (2) mandible movement, and (3) articulatory suppression, the order of which was randomised. In the articulatory suppression condition participants were required to silently repeat the word “the” every second for the duration of the SPiN task. In the mandible movement condition, participants were required to open and close their jaw every second for the duration of the SPiN task. In the passive listening conditions, participants completed the SPiN task alone without any mouth movement. The mandible movement condition aimed to provide a non-articulatory motor movement control condition (Stokes et al. 2019). The labels for these task conditions have been chosen to reflect the labels given in the Stokes et al. (2019) original paper; however, we note that although labelled ‘passive listening’, attentive listening is always required in order to perform a speech-perception task.

In the original paper by Stokes et al. (2019), the specific CVC stimuli employed were used to investigate whether or not the dual-task conditions impacted phoneme identification differently based on the place and mode of phoneme articulation. However, we did not seek to replicate this effect in the present study, instead we aimed to expand the findings to understand the impact of articulatory motor suppression on overall speech perception across age ranges.

### Procedure

After joining the video call, the researcher checked that the participant had headphones or earphones to wear during the SPiN task, and a working webcam. If the participant wore hearing aids, they were asked to remove them. All participants wore headphones or earphones for the duration of the experiment. The researcher provided a verbal explanation of the experimental procedure. If participants were able to share their computer screen, they were asked to do so, to allow the researcher to view the progression of the experiment. If this was not possible, due to technical difficulties, the participant verbally kept the researcher informed about the progression of the experiment. Regardless of whether or not screen sharing was possible, the researcher and participant remained on video call and the participant’s face was visible to the researcher at all times to allow sufficient monitoring of task adherence.

The participant accessed the experiment via an online link to Qualtrics, where they read the participant information sheet, provided consent, and then completed demographic questions, the SR-IQCODE, and the SSQ-12. Once the questionnaires were completed, the participant was automatically redirected to the Pavlovia hosting platform to complete the behavioural section of the experiment. The participant first viewed the experimental instructions. Then the participant was presented with an example of the speech stimuli, a word in quiet (at 76 dB SPL intensity), to allow them to adjust their computer or laptop volume to a loud but comfortable level. Following this, the participant completed four practice trials in which they listened to each CVC word in quiet, and selected the word they thought they heard from the image options on screen. Next, participants completed the first of the three conditions of the SPiN dual-task, either passive listening, mandible movement, or articulatory suppression. On-screen instructions informed the participant which task they were completing. The researcher also took time between each condition to ensure the participant understood the requirements, and allowed them to take a short break if needed. The researcher was present in the Microsoft Teams call for the duration of the experiment, on mute and with their camera off to prevent distraction, but observing to ensure participants completed the articulatory suppression and mandible movements as required. If participants forgot to do so, the researcher quickly reminded them. After completing all three conditions, the participant was debriefed and thanked for their participation and provided with course credits, or a 10 GBP shopping voucher.

### Data pre-processing and analysis

The outcome measure from the SPiN task was the statistic d' (‘d-prime’), which is a measure of the sensitivity at which the participant is able to correctly identify the target CVC word in noise. Initially, d' was compared across the three SPiN task conditions at the SNR at which younger adults achieved 75% correct performance in the passive listening condition. Therefore, in this first analysis, d' was compared across conditions relative to the 75% correct performance standard SNR in younger adults. We then conducted an exploratory analysis to compare d' achieved by younger adults and older adults across the three SPiN task conditions at the SNR at which either the younger or older adults achieved a 75% correct performance in the passive listening condition. Therefore, in this second analysis, d' was compared across conditions in each age group relative to the age-group-specific 75% correct SNR. In a two-alternative forced-choice (2AFC) task such as this one, 75% correct corresponds to a d' value of 0.95, and 50% correct corresponds to a d' of 0. In order to find a ‘benchmark’ SNR for the younger adults and older adults, the Palamedes MATLAB toolbox (Prins & Kingdom, 2018) was used to perform a maximum likelihood estimation of the best-fitting psychometric function for each participant.

The number of trials, and the number of correct responses at each tested SNR level in the passive listening condition, were fitted with a Gumbel (or log-Weibull) psychometric function, with slope and threshold as free parameters. The guess rate was fixed at 0.5, reflecting a 50% guess rate for a 2AFC task such as this, and the lapse rate was fixed at 0.02. The resulting psychometric function plots displayed the estimated proportion correct against continuous SNRs ranging from -16 to +16 dB. A total of five older adults (mean age = 75.4 years) did not achieve 75% correct in the passive listening condition, due to performing at floor at baseline. All five self-reported having age-related hearing loss, and four reported wearing hearing aids for both ears, which were removed during the task. These five participants were removed from further analyses.

Using the psychometric function data modelling the estimated proportion correct against continuous SNRs from the remaining participants, we extracted the estimated SNR at which each participant would achieve 75% correct performance. The mean SNR at which younger adults would obtain 75% correct was -0.66 dB (SD = 4.59), and the mean SNR at which older adults would obtain 75% correct was 5.27 dB (SD = 4.16).

For the primary analysis, we compared the d' values attained by all participants at -0.66 dB SNR (which gave a benchmark 75% performance for the younger adults), across all three SPiN dual-task conditions. As the primary test of our hypotheses, the reported *p*-values have been corrected to control for multiple comparisons. All additional tests, including follow-up tests on the main model, are considered exploratory and *p*-values are presented uncorrected.

In an exploratory analysis, we acquired the d' values obtained by younger adults at -0.66 dB SNR in each SPiN condition and the d' values obtained by older adults at 5.27 dB SNR in each SPiN condition; these values were then used to measure performance across the three conditions. This exploratory analysis allowed us to investigate age-group specific effects. This contrasts with the original analysis, which evaluated performance relative to the performance standard of younger adults’ performance in the passive listening condition.

To calculate d', the Palamedes MATLAB toolbox fitted Gumbel (or log-Weibull) psychometric functions, with slope and threshold as free parameters, a 0.5 guess rate, and 0.02 lapse rate, across all three SPiN dual-task conditions. The estimated proportion correct at -0.66 dB SNR or 5.27 dB SNR was extracted for all younger or older participants, respectively, and all conditions, and then converted into d' using built-in functions in Palamedes toolbox for 2AFC tasks. This resulted in three d' scores, one for each SPiN dual-task condition (passive listening, mandible movement, articulatory suppression), for all participants.

Occasionally, the psychometric function failed to fit the provided data. This occurred for two older adults in the mandible movement condition, and one older adult in the articulatory suppression condition. As such, there were three missing d' data points. However, as the data were statistically analysed using linear mixed effects models that are able to cope well with missing data points, these participants were not excluded from analysis.

R (R Core Team, 2022) and the ‘lme4’ package (Bates et al., 2015) were used to analyse the data using linear mixed effects models, which were conducted using the lmer() function. The models were used to examine the impact of the interaction between the fixed factors, SPiN dual-task condition (passive listening vs. mandible movement vs. articulatory suppression), age group (younger vs. older), and SSQ-12 scores, on the outcome d'. Participant was included as a random effect. This allowed for the comparison of d' across conditions, to understand the effects of articulatory motor suppression on speech perception, as well as the impact of age and subjective hearing ability.

The sample characteristics after the exclusion of five older adults performing at floor were as follows: Forty-six younger adults (M age = 20.17 years, SD = 1.61, 36 female), and 41 older adults (M age = 69 years, SD = 5.82, 21 female). Fourteen older adults self-reported that they had ARHL, seven of whom reported wearing hearing aids. The mean IQCODE-SR score was 2.08 (SD = 0.57) for younger participants and 3.12 (SD = 0.17) for older participants.

## Results

### Descriptive statistics

**Fig. 1:**
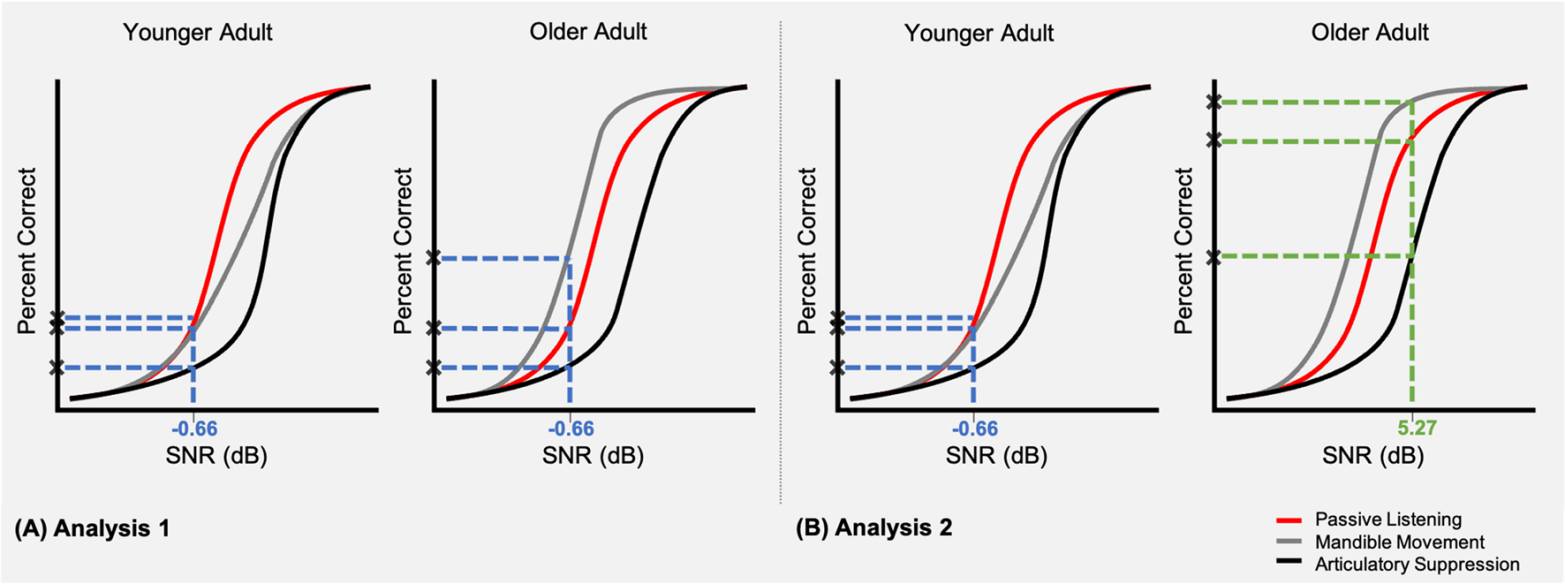
Schematic diagram to illustrate the analysis approaches. Diagram (**A**) shows an example younger adult and older adult performance comparison using the 75% correct younger adult performance standard (analysis version 1). Diagram (**B**) shows an example younger adult and older adult performance comparison using age-group performance standard (analysis version 2)

Table 1 displays descriptive statistics. Younger adults reported better hearing ability (SSQ-12 scores) than older adults, but this difference was not significant [*t*(71.43) = 0.87, uncorrected *p* = .38, Cohen’s *d* = 0.19]. For the primary analysis (see Fig. 1), younger adults showed better speech perception (greater d') than older adults during passive listening [*t*(80.83) = 5.24, uncorrected *p* < .001, Cohen’s *d* = 1.10], mandible movement [*t*(83.00) = 3.70, uncorrected *p* < .001, Cohen’s *d* = 0.80], and articulatory suppression [*t*(72.18) = 3.22, uncorrected *p* = .002, Cohen’s *d* = 0.67].

**Table 1:** Descriptive statistics (means and standard deviations) of scores on the SSQ-12, and of d' across the three experiment conditions, for older and younger participants

Age group	SSQ-12 score (SD)	Passive listening (SD)	Mandible movement (SD)	Articulatory suppression (SD)
		Mean d’ at the benchmark 75% correct SNR for younger adults during passive listening (-0.66 dB SNR)		
Younger	7.38 (1.03)	1.02 (0.37)	0.90 (0.33)	0.86 (0.46)
Older	7.14 (1.44)	0.66 (0.26)	0.66 (0.28)	0.61 (0.26)
		Mean d' at the benchmark 75% correct SNR for older adults during passive listening (5.27 dB SNR)		
		1.05 (0.43)	0.95 (0.40)	0.89 (0.43)

Means and standard deviations (SDs) before the removal of influential outliers

In an exploratory analysis (see Fig. 1), d' was equivalent across age groups during passive listening, as it reflects the age-group-specific 75% performance standard. There were no significant age group differences in d' during mandible movement [*t*(74.03) = -0.63, uncorrected *p* = .53, Cohen’s *d* = 0.140], or articulatory suppression [*t*(83.48) = -0.41, uncorrected *p* = .68, Cohen’s *d* = 0.09].

### Linear mixed effects models

The linear mixed effects model was: d' ~ SPiN dual-task condition * age group * SSQ-12 score + (1 | participant). The data met assumptions for linearity, homoscedasticity, and normality of residuals, and there was no multicollinearity among the variables (variance inflation factors ≤ 1.02). Influential outliers were investigated using Cook’s distance; 14 data points with a Cook’s distance > 4/*n*, where *n* is the total number of data points (*n* = 261) were removed from the analysis.

#### Analysis 1: d’ comparison relative to younger adult baseline

To justify inclusion of random effects, the full model was compared to an alternative model without the random effect of participant (i.e., d' ~ SPiN dual-task condition * age group * SSQ-12 score). The lower Akaike Information Criterion (AIC; Akaike, 1998) indicated that the full model was a better fit (AIC = -20.92) than the alternative (AIC = 102.43). Further, the full model was compared to: a null model [d' ~ (1| participant)]; and a model with main effects only (d' ~ SPiN dual-task condition + age group + SSQ-12 score). The full model (AIC = -20.92) was a better fit compared to the null (AIC = 22.83) and main effects model (AIC = -11.11).

Model results are reported in Table 2. *P* values are Bonferroni-Holm corrected across seven tests. The fixed effects explained 22.1% of the variance in the data (marginal R^2^ = .221), and 77.5% was explained by both fixed and random effects (conditional R^2^ = .775).

**Table 2:** Linear mixed model output detailing the statistical contribution of each fixed-effect predictor and interaction to the outcome of d’

Full model: d’ ~ SPiN dual-task condition * age group * SSQ-12 score + (1 | participant)						
Fixed effects:						
Predictor	Sum of squares	Mean square	Denominator DF	*F*	*p*	*Adj. p*
Age group	0.04	0.04	88.49	1.86	.176	.527
SPiN dual-task condition	0.16	0.08	162.40	3.48	.033	.133
SSQ-12 scores	0.04	0.04	88.73	1.73	.191	.526
Age group * SPiN dual-task condition	0.20	0.10	162.40	4.42	.014	.068
Age group * SSQ-12 scores	0.01	0.01	88.73	0.50	.483	.527
SPiN dual-task condition * SSQ-12 scores	0.27	0.14	162.55	5.87	.003	**.024**
Age group * SPiN dual-task condition * SSQ-12 scores	0.27	0.13	162.55	5.75	.004	**.024**

This table shows the analysis of variance output for the linear mixed effects model utilising the anova() function from the ‘lmerTest’ package, for fixed-effect terms using the Satterthwaite method for estimating denominator degrees of freedom. Adj. *p* values are corrected using Bonferroni-Holm across the number of predictors in the model

There were no significant main effects of: age group [*F*(1,88.49) = 1.86, adjusted *p* = .176], SPiN dual-task condition [*F*(2,162.40) = 4.42, adjusted *p* = .068], nor SSQ-12 scores [*F*(1,88.73) = 1.73, adjusted *p* = .526]. Nor were there significant interactions between age group and SPiN dual-task condition [*F*(2,162.40) = 4.41, adjusted *p* = .068], or age group and SSQ-12 scores [*F*(1,88.73) = 0.50, adjusted *p* = .527]. However, we observed a significant two-way interaction between SPiN dual-task condition and SSQ-12 scores on d' [*F*(2,162.55) = 5.87, adjusted *p* = .024], and a significant three-way interaction between age group, SPiN dual-task condition, and SSQ-12 scores on d' [*F*(2,162.55) = 5.75, adjusted *p* = .024].

#### SPiN dual-task condition and SSQ-12 scores interaction

There was a significant interaction between SSQ-12 scores and SPiN dual-task condition on speech perception (d'). Exploratory correlation analyses revealed that across all participants, there was a significant relationship between better self-rated hearing ability (higher SSQ-12 scores) and better speech perception during passive listening [*r*(80) = .24, uncorrected *p* = .027] and during mandible movement [*r*(79) = .27, uncorrected *p* = .014], but not during articulatory suppression [*r*(79) = .03, uncorrected *p* = .785].

#### SPiN dual-task condition, SSQ-12 scores, and age-group interaction

To examine the significant three-way interaction between age group, SSQ-12, and SPiN dual-task condition, two exploratory linear mixed models were conducted with the data split by age group [i.e., d' ~ SPiN dual-task condition * SSQ-12 score + (1 | participant)].

For younger adults, there was a significant main effect of SPiN dual-task condition [*F*(2,79.99) = 4.36, uncorrected *p* = .016] and a significant interaction between SSQ-12 score and SPiN dual-task condition on d' [*F*(2,80.10) = 6.41, uncorrected *p* = .003]. Post hoc comparisons indicated that speech perception was poorest during articulatory suppression compared to both mandible movement [β = 0.11, *t*(78.6) = 3.07, uncorrected *p* = .003] and passive listening [β = 0.19, *t*(78.6) = 5.17, uncorrected *p* < .001], and speech perception was poorer during mandible movement compared to passive listening [β = 0.08, *t*(78.0) = 2.21, uncorrected *p* = .030]. Post hoc exploratory correlations between subjective hearing ability and speech perception were not statistically significant.

**Fig. 2:**
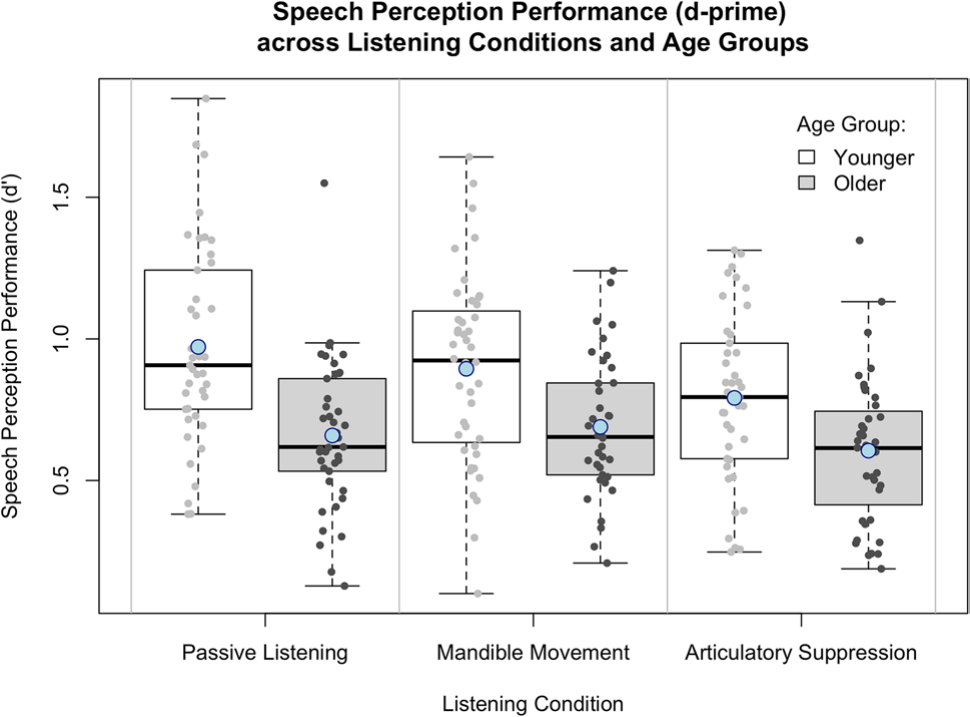
Speech perception performance, indicated by d’, across younger and older adults for the three listening conditions (passive listening, mandible movement, and articulatory suppression). Higher values indicate better performance. Error bars represent standard error. Means are indicated by blue circles

**Fig. 3:**
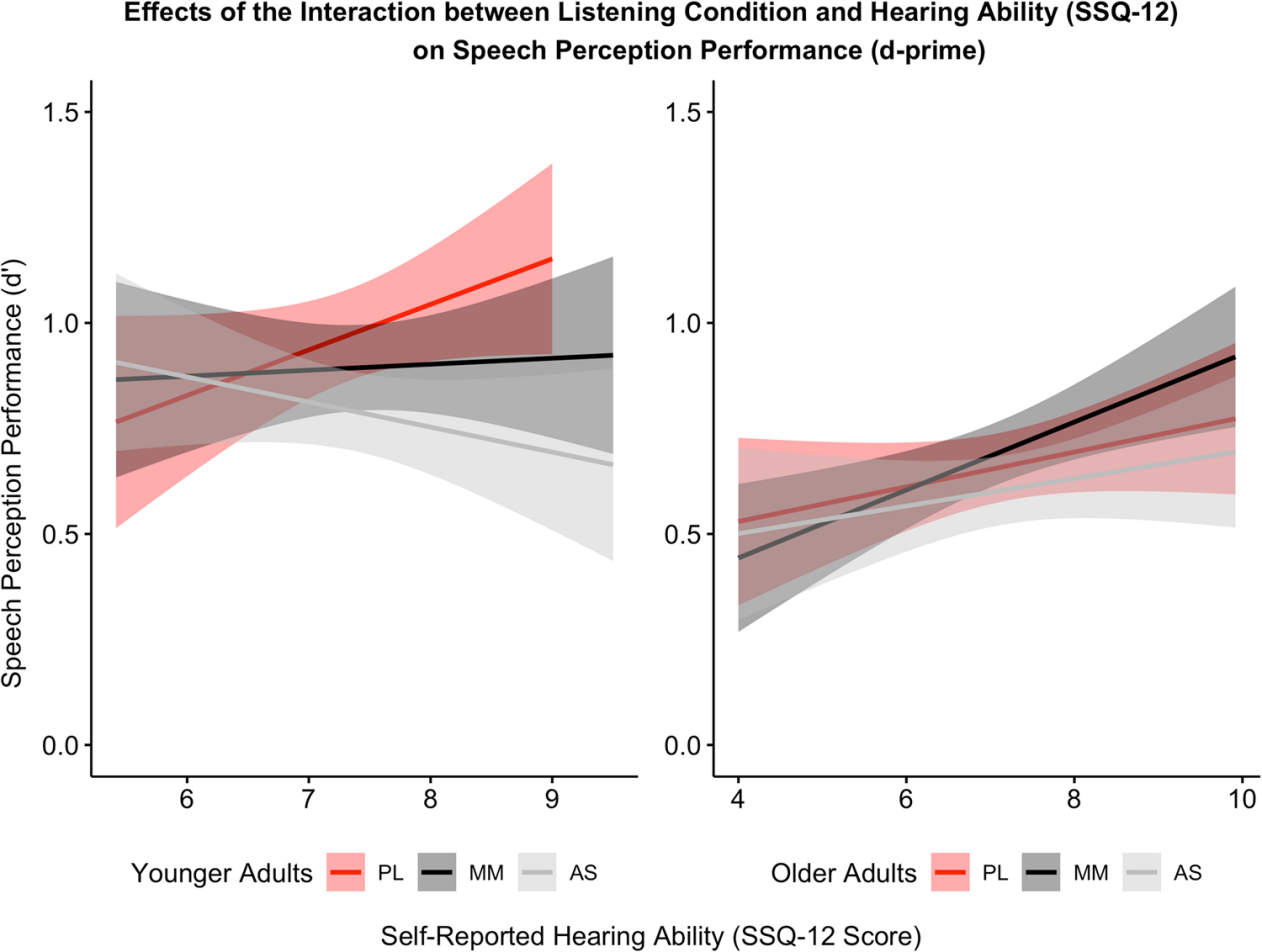
Speech perception performance, indicated by d', correlated with self-reported hearing ability, indicated by SSQ-12 scores, across the three listening conditions (PL = passive listening, MM = mandible movement, and AS = articulatory suppression) for younger (**left side**) and older (**right side**) adults. Higher values indicate better performance, and better self-reported hearing ability

**Fig. 4:**
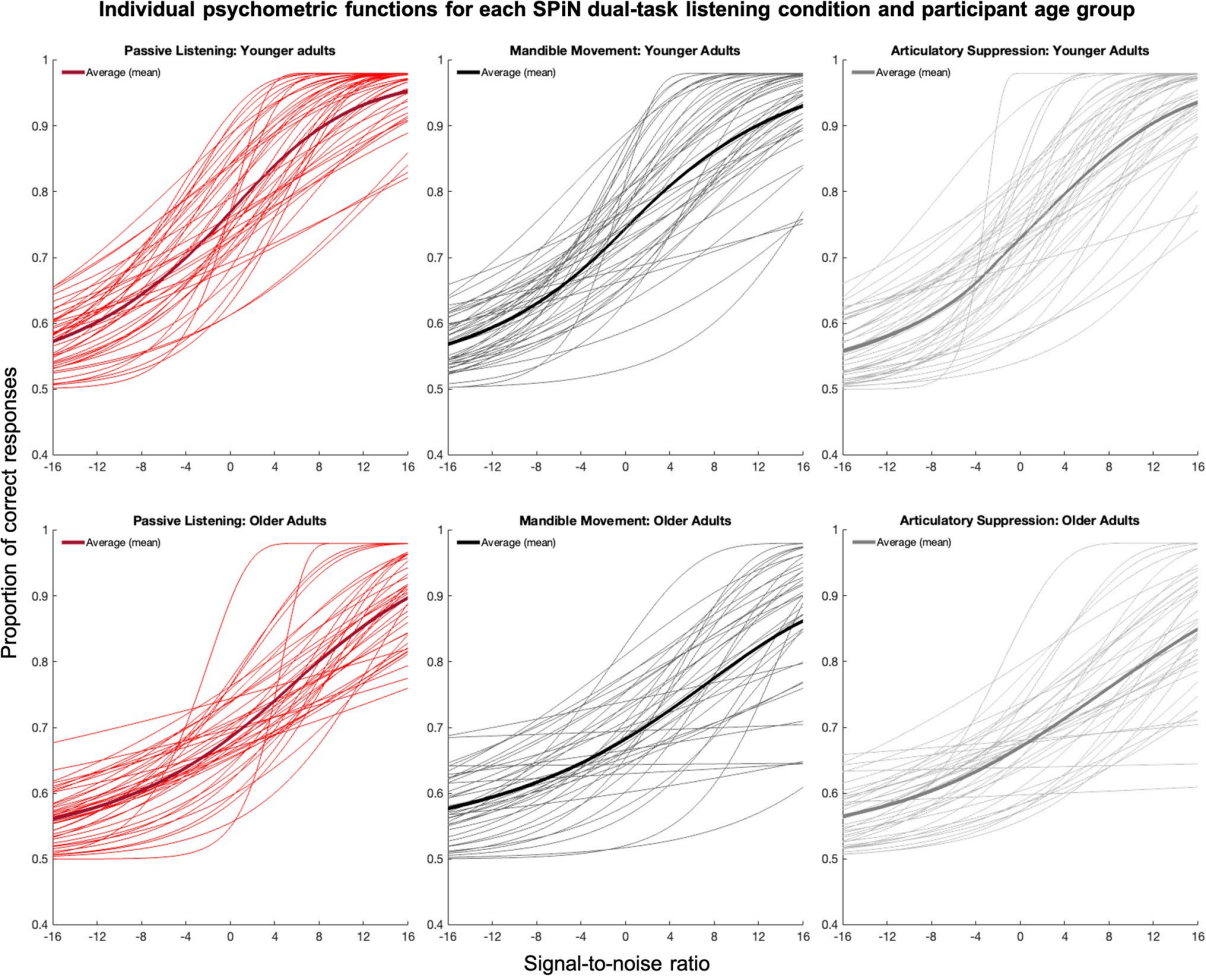
The individual psychometric functions illustrating the proportion of correct responses at signal-to-noise ratios from -16 dB to + 16 dB, in each SPiN dual-task listening condition, for older and younger adults

For older adults, there were no significant effects of SPiN dual-task condition [*F*(2,73.33) = 0.56, uncorrected *p* = .575]; SSQ-12 scores [*F*(1,38.77) = 3.95, uncorrected *p* = .054]; nor of the interaction between SPiN condition and SSQ-12 scores [*F*(2,73.37) = 0.94, uncorrected *p* = .394] on d’ Figs. 2, 3 and 4.

#### Exploring the impact of hearing and cognitive status

We explored if including hearing status (i.e., self-reported age-related hearing loss vs. no hearing loss) or cognitive status (i.e., SR-IQCODE) in the linear mixed effects model would explain any of the variation in speech perception (d’).

This model was conducted on the older adult data, as all younger adults had normal hearing: d' ~ SPiN dual-task condition * hearing status + (1 | participant). There were no significant effects of hearing status [*F*(1,38.91) = 0.32, uncorrected *p* = .575], nor of the interaction between hearing status and SPiN dual-task condition [*F*(2,73.48) = 1.00, uncorrected *p* = .374] on d’. To investigate the impact of cognitive status, we incorporated SR-IQCODE as a covariate: d’ ~ SPiN dual-task condition * age group * SSQ-12 score + SR-IQCODE score + (1 | participant), but it did not significantly impact d’ [*F*(1, 87.46) = 0.09, uncorrected *p* = .768]. Further, goodness-of-fit criteria suggested that incorporating SR-IQCODE did not provide a better explanatory model (AIC = -19.01) than the full model (AIC = -20.93).

#### Analysis 2: Exploratory d’ comparison relative to age-group baseline

We conducted an exploratory model to compare d' across SPiN conditions relative to an age-group-specific baseline during passive listening. There were no significant main effects of: age group [*F*(1,86.05) = 0.98, uncorrected *p* = . 324]; SPiN dual-task condition [*F*(2,160.12) = 1.73, uncorrected *p* = .181]; SSQ-12 scores [*F*(1,86.27) = 3.62, uncorrected *p* = .060], nor a significant interaction between age group and SSQ-12 scores [*F*(1,86.27) = 1.49, uncorrected *p* = .226]. However, we observed significant two-way interactions between SPiN dual-task condition and age group [*F*(2,160.12) = 6.02, uncorrected *p* = .003] and SSQ-12 scores and listening condition [*F*(2,160.26) = 3.68, uncorrected *p* = .027] on d', and a three-way interaction between age group, SPiN dual-task condition, and SSQ-12 scores groups [*F*(2,160.26) = 6.66, uncorrected *p* = .002] on d’.

#### SPiN dual-task condition, SSQ-12 scores, and age-group interactions

To examine the interaction effects, exploratory models were conducted with the data split by age group. For younger adults, there was a significant main effect of SPiN dual-task condition [*F*(2,80.88) = 5.42, uncorrected *p* = .006] and interaction effect between SPiN dual-task condition and SSQ-12 score on d' [*F*(2,80.97) = 7.85, uncorrected *p* = .001]. Post hoc comparisons indicated that speech perception was poorest during articulatory suppression compared to mandible movement [β = 0.11, *t*(79.5) = 3.03, uncorrected *p* = .003] and passive listening [β = 0.21, *t*(79.4) = 5.44, uncorrected *p* < .001], and speech perception was poorer during mandible movement compared to passive listening [β = 0.09, *t*(78.8) = 2.51, uncorrected *p* = .014]. Further exploratory correlations revealed that better subjective hearing was significantly associated with better speech perception during passive listening [*r*(40) = .32, uncorrected *p* = .040], but not during mandible movement [*r*(42) = .04, uncorrected *p* = .782] or articulatory suppression [*r*(39) = -.19, uncorrected *p* = .223].

**Fig. 5:**
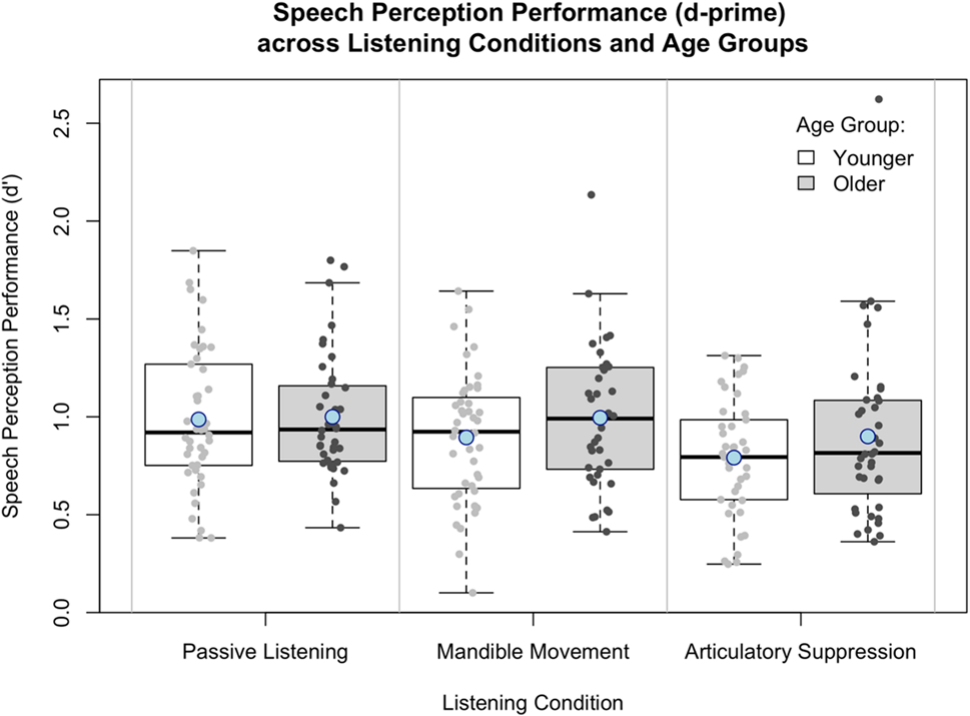
Linear mixed model analysis version 2: Speech perception performance, indicated by d', across younger and older adults for the three listening conditions (passive listening, mandible movement, and articulatory suppression). Higher values indicate better performance. Error bars represent standard error. Means are indicated by blue circles

For older adults, there were no significant effects of SPiN dual-task condition [*F*(2,70.28) = 1.58, uncorrected *p* = .212], nor of the interaction between SPiN condition and SSQ-12 scores [*F*(2,70.35) = 1.22, uncorrected *p* = .301] on d'. There was a significant effect of SSQ-12 scores [*F*(1,36.66) = 6.07, uncorrected *p* = .019] on d’. Exploratory correlations revealed that better subjective hearing was significantly associated with better speech perception during mandible movement [*r*(35) = .55, uncorrected *p* < .001], but not during passive listening [*r*(38) = .23, uncorrected *p* = .153], or articulatory suppression [*r*(38) = .26, uncorrected *p* = .101] Fig. 5.

## Discussion

The data did not support H1, as there was no main effect of dual-task SPiN condition on speech perception. Descriptive statistics show that speech perception, relative to younger adults’ baseline, was poorest during articulatory suppression (M = 0.70, SD = 0.29), followed by mandible movement (M = 0.80, SD = 0.31), and best during passive listening (M = 0.82, SD = 0.35), but these differences were not significant.

The data partially supported H2, as we observed significant interactions between age group, SPiN dual-task condition, and SSQ-12 scores on speech perception. In both versions of the linear mixed model analysis younger adults, but not older adults, showed significant differences in d' across task conditions. Specifically, in younger adults, speech perception was significantly poorest during articulatory suppression compared to passive listening and mandible movement. Younger adults’ performance was also significantly worse during mandible movement compared to passive listening. In older adults, there were no significant differences in performance across conditions. This finding is partly consistent with the Stokes et al. (2019) paper on younger adults only. Stokes et al. found that speech perception, indicated by the 75% correct threshold, was significantly poorer in articulatory suppression versus passive listening. However, the authors did not find any differences between articulatory suppression and other non-speech movement conditions.

The data did not support H3, as there was no interaction between speech perception and subjective hearing ability in older adults. There was a significant interaction between speech perception and subjective hearing ability in younger adults. However, the post hoc exploratory correlations were not consistently significant.

### Auditory-motor compensation or auditory-motor decline?

These data appear to support the auditory-motor decline hypothesis, which posits that for older adults, with likely poorer auditory function, there is reduced recruitment of articulatory motor resources during speech perception (Panouillères & Möttönen, 2018). In younger adults, with likely better auditory function, articulatory motor resources may serve as a compensatory tool during difficult listening. Indeed, younger adults in this study showed poorer speech perception when their articulatory motor resources were occupied, corroborating findings from the original paper that we sought to partially replicate and extend (Stokes et al., 2019). Occupying speech motor resources through repetitive speech movements in this study may have reduced the resource capacity available for speech perception, negatively impacting performance.

However, no such pattern was observed in the older adults, who were worse overall relative to younger adults. Our data indicate that motor resources may be reduced in ageing, alongside reduced auditory resources. Due to limited resources, the speech motor system may not be able to support speech perception to the same extent as in younger listeners. Indeed, previous researchers have found that activation of speech motor resources is reduced in older adults with hearing loss (Panouillères & Möttönen, 2018) and older adults with poorer hearing acuity (Nuttall et al., 2022). According to the auditory-motor decline hypothesis, age-related atrophies in the peripheral and central auditory system reduce information input to the articulatory motor cortex via the dorsal stream, thus reducing articulatory motor activity (Panouillères & Möttönen, 2018). Reduced motor processing may also have implications for successful turn-taking during everyday conversation by predicting the conversational partner’s next turn (Garrod & Pickering, 2015; Scott et al., 2009). Reduced motor processing may lead to imprecise estimation of turning-taking, further negatively impacting social interaction for older adults with hearing loss.

### Limitations and future directions

Due to online testing, assessing clinical hearing acuity was not possible. Instead, participants self-reported hearing ability using the SSQ-12. The SSQ-12 showed no age-related differences, therefore the measure may capture a different dimension of hearing, such as self-efficacy, instead of age-related hearing difficulties. Considering that 41.3% of the older participants in our study self-reported having ARHL, central and peripheral auditory atrophies characteristic of hearing loss may underpin our findings, i.e., reduced auditory-motor integration reduces the facilitatory speech motor resources for speech perception. The percentage of older individuals with clinical hearing loss in this study may have been higher than estimated, and future work should seek to evaluate this in a laboratory setting using audiometry. Indeed, other limitations of online auditory research should be acknowledged, including reduced control over the presentation of the auditory stimuli due to differences in computer or audio equipment. However, through employing a repeated-measures design in which all participants completed each SPiN dual-task condition, any variances should be accounted for and affect each condition equally.

Reduced speech perception during articulatory suppression in younger adults may be related to increased cognitive demands that are not necessarily specific to articulatory motor resources. In this study, articulatory suppression is assumed to utilise speech motor resources. However, cognitive resources may also be involved in task switching or working memory processes, which are also important for speech perception. Further, as noted by Stokes et al. (2019), silent articulatory suppression may also generate activity related ‘auditory imagery’, not only articulatory speech-motor activity, which may have contributed to the disruptive effects of this condition.

Further, the finding that older adults showed similar performance across dual-task conditions may indicate that the task was too challenging, which can lead to withdrawal of effort (Slade et al., 2021). Future studies should consider both difficulty and success importance (i.e., the cost-benefit analysis of investing effort), as these motivational factors may be different in younger and older listeners (Ennis et al., 2013). In combination with the data presented here, such work would inform how ageing and hearing loss impact resource allocation during speech perception, providing direction for future rehabilitation interventions for ARHL.

## Data Availability

The data associated with this manuscript are openly available in the OSF repository at https://osf.io/y79n6/.

## Abbreviations

ARHL: Age-Related Hearing Loss
TMS: Transcranial Magnetic Stimulation
fMRI: Functional Magnetic Resonance Imaging
SD: Standard Deviation
SR-IQCODE: Self-Report version of the Informant Questionnaire on Cognitive Decline
SSQ-12: Spatial Qualities of Hearing Scale
SPiN: Speech Perception in Noise
CVC: Consonant-Vowel-Consonant
SNR: Signal-to-Noise Ratio
2AFC: Two-Alternative Forced-Choice
AIC: Akaike Information Criterion
PL: Passive Listening
MM: Mandible Movement
AS: Articulatory Suppression
